# Explainable Artificial Intelligence to Predict the Water Status of Cotton (*Gossypium hirsutum* L., 1763) from Sentinel-2 Images in the Mediterranean Area

**DOI:** 10.3390/plants13233325

**Published:** 2024-11-27

**Authors:** Simone Pietro Garofalo, Anna Francesca Modugno, Gabriele De Carolis, Nicola Sanitate, Mesele Negash Tesemma, Giuseppe Scarascia-Mugnozza, Yitagesu Tekle Tegegne, Pasquale Campi

**Affiliations:** 1 Council for Agricultural Research and Economics, Research Center for Agriculture and Environment, Via Celso Ulpiani, 5, 70125 Bari, Italy; 2 Biocities Facility, European Forest Institute, Via Manziana 30, 00189 Roma, Italy; 3 Circular Bioeconomy Alliance, 71 Queen Victoria Street, London EC4V 4BE, UK

**Keywords:** drought stress, Gossypium, machine learning, satellite, remote sensing, Optuna

## Abstract

Climate change and water scarcity bring significant challenges to agricultural systems in the Mediterranean region. Novel methods are required to rapidly monitor the water stress of the crop to avoid qualitative losses of agricultural products. This study aimed to predict the stem water potential of cotton (*Gossypium hirsutum* L., 1763) using Sentinel-2 satellite imagery and machine learning techniques to enhance monitoring and management of cotton’s water status. The research was conducted in Rutigliano, Southern Italy, during the 2023 cotton growing season. Different machine learning algorithms, including random forest, support vector regression, and extreme gradient boosting, were evaluated using Sentinel-2 spectral bands as predictors. The models’ performance was assessed using R^2^ and root mean square error (RMSE). Feature importance was analyzed using permutation importance and SHAP methods. The random forest model using Sentinel-2 bands’ reflectance as predictors showed the highest performance, with an R^2^ of 0.75 (±0.07) and an RMSE of 0.11 (±0.02). XGBoost (R^2^: 0.73 ± 0.09, RMSE: 0.12 ± 0.02) and AdaBoost (R^2^: 0.67 ± 0.08, RMSE: 0.13 ± 0.02) followed in performance. Visible (blue and red) and red edge bands were identified as the most influential predictors. The trained RF model was used to model the seasonal trend of cotton’s stem water potential, detecting periods of acute and moderate water stress. This approach demonstrates the prospective for high-frequency, non-invasive monitoring of cotton’s water status, which could support smart irrigation strategies and improve water use efficiency in Mediterranean cotton production.

## 1. Introduction

Climate change is deeply affecting agriculture through a variety of mechanisms, bringing significant challenges to agricultural sustainability. Rising global temperatures, altered precipitation patterns, and the increased frequency of extreme weather events (such as droughts, floods, and heat waves) are transforming the agricultural landscape, leading to reduced crop yields and compromised production [1,2]. Adaptation strategies, such as changing planting times, crop rotation, agroforestry, development of drought-resistant crops and varieties, and the use of strategies to increase water productivity through deficit irrigation, are essential to mitigate these impacts and ensure sustainable agricultural productivity [1,3]. Climate change could significantly affect the yield and fiber quality of cotton (*Gossypium hirsutum* L., 1763) [4]. Given its worldwide importance, these potential impacts are particularly concerning. Although cotton is generally considered a drought-resistant crop, continuous water stress can lead to reduced fiber quality; moreover, its monoculture cultivation requires high inputs, especially water and chemicals, to maximize productivity [5,6]. The negative effect of drought stress on cotton yield, exacerbated by climate change’s impact, depends on several factors, such as when water stress occurs (phenology), its intensity, and the cotton cultivar [7,8]. For instance, thermal and water stresses could lead to lower boll volume and dry matter, and reduced fiber length and strength [9,10]. In this context, having methods that allow the rapid monitoring of the water status of cotton could help avoid such negative effects, and improve water saving and water productivity.

Traditional methods for assessing cotton’s water status include measurements of soil moisture, leaf water potential, stomatal conductance, and stem water potential. Soil moisture sensors, such as tensiometers and capacitance probes, provide data on the water content of the soil profile but may not accurately reflect the plant water status due to factors like root distribution, soil heterogeneity, and depth [11]. Leaf water potential, measured using a pressure chamber technique [12], offers insight into the water tension within the leaves but can be influenced by environmental conditions and diurnal variations [13]. Stem water potential has emerged as a more stable and reliable indicator of plant water status, integrating the overall water balance of the plant [14]. The measurement involves enclosing a leaf in a foil bag for equilibration before determining the water potential using a pressure chamber [14]. This method provides a direct assessment of the plant’s hydration status; research has shown that the stem water potential is highly sensitive to water stress, making it a reliable parameter for irrigation management [15]. However, the technique is labor-intensive and time-consuming, and requires specialized equipment and expertise, limiting its practicality for large-scale or frequent monitoring [16]. Therefore, taking a sufficient amount of stem water potential measurements to be representative within the field and throughout the growing season could be difficult for irrigation scheduling. The integration of remote sensing and machine learning techniques could allow the fast monitoring of several parameters that are useful for crop irrigation management, including stem water potential [17,18]. Remote sensing technology enables the observation and analysis of the Earth’s surface characteristics by measuring the radiation reflected or emitted from objects, without direct physical contact [19]. This data collection is mainly achieved through satellites, aircraft, and, increasingly, unmanned aerial vehicles (UAVs), providing accurate results at variable costs depending on the platform used. In recent years, the agricultural sector has seen a significant increase in the adoption and application of these remote-sensing technologies [19]. Sentinel-2 is a mission of the Copernicus program of the European Space Agency; it involves two satellites, 2A and 2B, with a revisit time of 5 days [20]. Sentinel-2 data can enhance precision agriculture and crop monitoring by providing high-resolution multispectral images that support multiple agricultural applications. For instance, Hassanpour et al. [21] used Sentinel-2 time series to monitor leaf area index, fractional vegetation cover, and canopy water content at the field scale. Also, Sentinel-2 imagery can be used to estimate important agronomic parameters such as the aboveground biomass of crops [22]. In crop monitoring and analysis, remote sensing data are utilized through three approaches: parametric, non-parametric, and physically based. Parametric approaches study the direct relationship between remote sensing data and crop traits but require certain statistical assumptions to be satisfied (e.g., linear regression); non-parametric approaches (e.g., machine learning algorithms, such as random forest) can capture non-linear and complex relationships, and can also handle non-normal distributions; physically based approaches are based on physical criteria, but their intricate nature often limits their practical application [23]. The use of machine learning algorithms is continuously increasing in agriculture, especially in remote sensing applications [24]. Machine learning utilizes statistical models and algorithms to analyze and identify patterns in data, in order to make predictions or decisions based on that data [25]. Machine learning in agriculture can help in monitoring crop parameters, improving productivity and resource efficiency [26]. For example, Narmilan et al. [27] compared different algorithms to predict sugarcane’s chlorophyll content, obtaining good results with the extreme gradient boosting algorithm. Choudhary et al. [28] used random forest to map rice yield from Sentinel-2 imagery.

For monitoring cotton’s water status, different studies investigated the use of remote sensing data. For instance, Bian et al. [29] used a drone to measure cotton’s crop water stress index under different irrigation treatments. Ballester et al. [30] used drone imagery to monitor the effects of water stress on cotton through the computation of vegetation indices. While different studies have utilized remote sensing and machine learning to assess cotton traits, few studies have investigated the integration of remote sensing data from satellite and machine learning techniques in monitoring cotton’s stem water potential, especially in the Mediterranean area [31]. 

This study aimed to investigate the feasibility of the integration of remote sensing and machine learning to predict cotton’s stem water potential in the Mediterranean area (southern Italy). Different machine learning algorithms have been tested and compared, with the aim of identifying the best-performing one for estimations of cotton’s water status. Furthermore, in this study, explainable machine learning techniques have been used to understand the role of the different variables in predicting stem water potential, thus providing insights into the driving factors influencing the model’s decision.

## 2. Materials and Methods

### 2.1. Experimental Area and Crop Management

**Figure 1:**
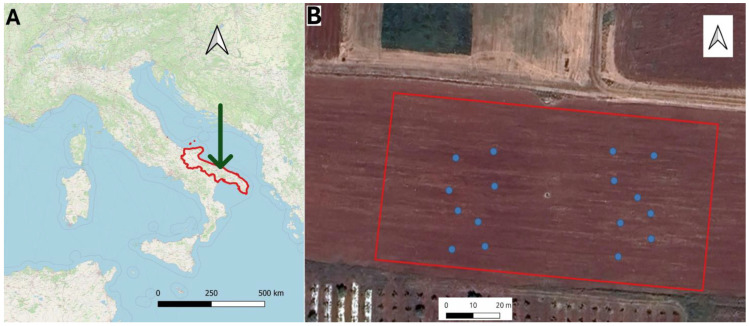
(**A**) Location of the experimental farm in Rutigliano (green arrow), Apulia region (red boundaries). (**B**) The cultivation area of cotton with the sampling points where stem water potential was determined (blue rings).

The trial was carried out during the 2023 season in Rutigliano in the south of Italy within the experimental farm belonging to the Council for Agricultural Research and Economics (40°59′ N; 17°01′ E; 147 m a.s.l) (Figure 1A). The climate is Mediterranean, with hot summers and warm and not very cold winters. According to the Köppen and Geiger classification, the climate in the region is categorized as CSa (hot-summer Mediterranean climates) [26]. Average annual precipitation is 535 mm, mainly concentrated in autumn and winter, and almost absent in the summer period; therefore, most species can be successfully grown in the spring–summer period in this area only by providing irrigation water supply. The agrometeorological data were recorded by the agrometeorological station installed within the farm.

Cotton (*Gossypium hirsutum* L., 1763; cultivar ST402, Pioneer) was grown on an area of 0.8 ha (Figure 1B). The soil of the field was classified as clay loam (USDA classification) and Lithic Ruptic Rhodoxeralf (FAO classification). Cotton was sown on day of the year (DOY) 150 with a plant density of 10 m^−2^; it was fertilized with 50 kg ha^−1^ of N, 25 kg ha^−1^ P_2_O_5_, and 20 kg ha^−1^ K_2_O through fertilization. Irrigation was managed to replace the total amount of water lost through the evapotranspiration of the crop (ETc), which was quantified following the methodology indicated by Allen et al. [32]. Tabulated crop coefficients were adopted, namely Kc_ini_ = 0.15, Kc_med_ = 1.10, and Kc_end_ = 0.50; a depletion fraction value of 0.50 was also used. Corrections to Kc_ini_ (for precipitation events) and to Kc_med_ and Kc_end_ for climatic conditions and crop height were carried out following the abovementioned Allen methodology. A drip irrigation system was adopted with a flow rate of 4 L h^−1^ per dripper applied to a 0.30 m dripper.

Cotton was harvested by hand during the first weeks of October.

### 2.2. Water Status of Cotton

The water status of the cotton was determined by measuring the stem water potential (SWP; MPa). SWP was determined during the season on cotton plants across 16 random points (Figure 1B). Measurements were carried out between 11.00 and 13.00 h solar. Before SWP determinations, adult and fully expanded leaves were placed into aluminum bags for 60 min, and then a Scholander-type pressure chamber connected to a cylinder containing nitrogen (Soil Moisture Equipment Corp., Santa Barbara, CA, USA) was used to measure SWP, by insufflating nitrogen until reaching equilibrium [3]. SWP was measured on DOYs 194, 199, 204, 209, 224, and 234, according to the time of Sentinel-2 crossing over the area.

### 2.3. Satellite Images

Images from Sentinel-2 were used in this study. Sentinel-2 satellites provide multispectral images with 13 spectral bands (S2-Bs) and high resolution (from 10 m to 60 m, depending on the band) [33]. Bands B01 (coastal aerosol), B09 (water vapor), and B10 (SWIR—cirrus) were not used in this study because they are not usually used for agricultural purposes [33]; all the remaining S2-Bs (B02, B03, B04, B05, B06, B07, B08, B8A, B11, and B12) were used as predictors in the analyses. Before reflectance data extraction, the Sentinel-2 images were resampled at a spatial resolution of 10 m using the library “rasterio” within the Python environment [34] (Python version: 3.11.5 64-bit) through the nearest neighbor method. For each field point where cotton’s SWP was measured, the reflectance values of the corresponding pixels were extracted for all the S2-Bs considered, using QGis (v. 3.28.15-Firenze for Windows) and the plug-in “Value Tool” (v. 3.0.19). This process was repeated across all 16 field points for each measurement date, building the dataset needed for the analyses, where SWP was considered the target variable and the S2-Bs as predictors [35]. All the Sentinel-2 images (*n* = 6) were downloaded from the online tool of Copernicus [36] as Level 2A products (atmospherically corrected surface reflectance).

### 2.4. Machine Learning Analyses

Modeling approaches compared in this study involved different machine learning algorithms: adaptive boosting (AdaBoost), support vector regressor (SVR), least absolute shrinkage and selection operator (Lasso), ridge regression (ridge) partial least square regression (PLSR), random forest (RF), and extreme gradient boosting (XGBoost). AdaBoost is an ensemble learning technique that combines multiple weak learners (i.e., decision trees) to enhance prediction; the central idea of AdaBoost is the iterative regulation of the weights of the training samples based on the errors of the previous models, thus focusing more on the hard-to-predict instances in subsequent iterations [37]. SVR is a popular machine learning technique used to solve regression problems by finding a hyperplane that minimizes the prediction errors; it is based on support vectors (the data points closest to the hyperplane) that are used to define the position and orientation of the hyperplane [38]. Lasso regression is a linear regression technique incorporating L1 regularization to improve the accuracy and interpretability of the model. Introduced by Robert Tibshirani [39], Lasso adds a penalty equal to the absolute value of the coefficients, promoting sparsity and reducing some coefficients to zero. This feature allows for efficient variable selection, making Lasso particularly useful in high-dimensional datasets where multicollinearity may be present [40]. Ridge regression is a regularization technique used to address multicollinearity in multiple regression models by adding a penalty to the loss function; this technique improves the estimation of coefficients by shrinking them towards zero, which reduces variance at the cost of introducing some bias, thereby enhancing the model’s performance [41,42]. PLSR is a multivariate method for modeling relationships between sets of observed variables when the predictors are highly collinear or when the number of predictors exceeds the number of observations. PLSR combines the characteristics of principal component analysis and multiple regression, allowing the extraction of latent variables that capture the most variance in the predictor variables and are also relevant to the prediction of the response variable [43,44]. RF is another supervised ensemble learning technique. RF improves regression by combining several decision trees to enhance the model’s performance and its generalization [35,45]. RF builds multiple randomized decision trees and averages the predictions of the single trees; this ensemble method improves the stability and accuracy of the model [46]. XGBoost is a highly efficient and scalable implementation of gradient boosting, a machine learning technique that combines predictions from multiple weak models (decision trees) to create a more powerful predictive model. Developed by Tianqi Chen and Carlos Guestrin [47], XGBoost has gained popularity for its performance and ability to handle large-scale data. XGBoost builds decision trees sequentially, and each tree is trained to correct the errors made by previous trees, focusing on the residuals of the predictions. This sequential training allows XGBoost to optimize the model iteratively, adjusting the predictions based on the performance of the ensemble as a whole [48,49]. 

All the analyses were carried out within the Python environment using the scikit-learn library (v. 1.3.0) (www.scikit-learn.org). For each model, fine-tuning of the hyper-parameters was carried out using the “Optuna” library (v. 3.6.1). Optuna consists of an automatic hyper-parameter optimization framework that efficiently researches the parameter space to identify the best-performing configuration. In this process, 50 optimization trials were performed for each model, using a combination of Bayesian optimization with the Tree-structured Parzen Estimator (TPE) sampler [50]. Table 1 reports the hyper-parameters fine-tuned for each model using Optuna.

To ensure robust and unbiased model evaluation, the dataset was randomly divided five times into training and testing sets, with each split allocating 70% of the data for training and 30% for testing. This approach allows each model to be trained on different subsets of data and evaluated on distinct testing sets, providing a comprehensive assessment of the model’s performance and generalization capabilities. This random split approach is a common practice in machine learning to enhance the reliability of model comparisons [51]. With the optimized hyper-parameters, each model was trained on the 70% training subset from each of the five random splits. This training process enabled the models to learn patterns and relationships between the S2-Bs’ reflectance values and the cotton’s SWP. After training, the models were evaluated on the 30% testing subset corresponding to each split, using the ground truth data—the actual observed values not included in the training phase—to assess the predictive accuracy and performance. To evaluate and compare the models, the coefficient of determination (*R*^2^; Equation (1)) and root mean square error (RMSE; Equation (2)) were calculated as follows

(1) $$R^{2}=1-\left(\frac{SS_{res}}{SS_{tot}}\right)$$

(2) $$RMSE=\sqrt{\frac{1}{n}\sum_{i=1}^{n}{\left(S_{i}-O_{i}\right)}^{2}}$$

where SSres is the sum of the squares of the residuals, SStot is the total sum of the squares, S_i_ is the simulated values, O_i_ is the observed value, and *n* is the number of observations. The models’ performance parameters were compared using Tukey’s test (α = 0.05) following an analysis of variance (ANOVA) conducted on the results of the 5 random splits of the dataset. All the analyses were carried out using Spyder © IDE (v. 5.4.3 for Windows).

### 2.5. Machine Learning Inference and Explainability

Once the best model among the calibrated ones had been found, it was applied to the available (and cloud-free) Sentinel-2 images (*n* = 12) for the period from the full development of cotton plants (early July 2023) to the ripening stage (mid-September); then the simulated values of SWP for each image were used to model the temporal variability in the cotton’s water status during the season.

Machine learning explainability is becoming more widely recognized as a critical component in the development and spread of machine learning systems; as machine learning models gain in complexity, comprehension of their behavior and outcomes becomes essential for users, stakeholders, and policymakers [52,53]. In this research, two methods were used to explain the predictions: permutation importance and the SHapley Additive exPlanations (SHAP) method. Permutation importance is a technique for understanding which features are the most influential in the predictions of a machine learning model. The process involves calibrating a model on the original dataset, defining a performance baseline, and then randomly changing the values of a specific feature to interrupt its association with the target variable. The model is then applied to obtain predictions on this altered dataset, and the importance of the feature is determined by the difference in the model’s performance before and after the permutation [54]. SHAP applies the concept of “Shapley value” from cooperative game theory, which quantifies the average marginal contribution of a player among all possible coalitions [55]. In the field of machine learning, it is applied to clarify the importance of features in relation to a predicted variable, highlighting how each feature influences the prediction [56,57]. Partial dependence is another technique that increases models’ interpretability. It was calculated for the four most important variables (from permutation and SHAP analysis) to illustrate their relationship with the target [58].

## 3. Results

### 3.1. Field Data

**Figure 2:**
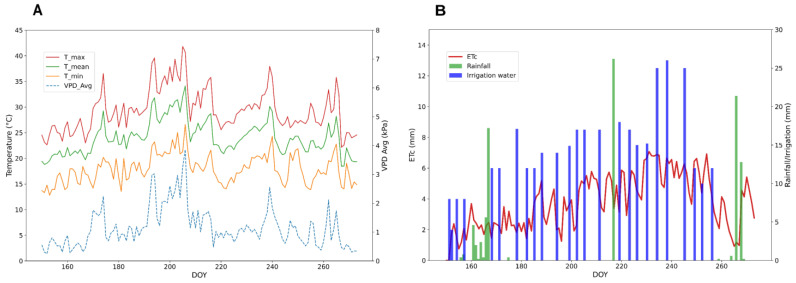
(**A**) The seasonal trend of average, maximum, and minimum temperatures, and vapor pressure deficit (VPD). (**B**) The seasonal trend of crop evapotranspiration (ETc), and the amount of rainfall and irrigation water applied.

In June, the average temperatures ranged between 18 and 29 °C, and a peak of maximum temperature of 36.56 °C occurred on DOY 174. July and August were the months with the highest temperatures recorded. During the second and third weeks of July, the average temperatures were stably above 25 °C, and the maximum temperatures reached 39.59 °C on DOY 194, 40.73 °C on DOY 204, and 41.83 °C on DOY 205. During August, temperatures were slightly lower than in July; nonetheless, the average temperatures were above 20 °C. In the second week of August, maximum temperatures were relatively low, ranging from 26 to 29 °C; they increased from DOY 233 (>~30 °C; 37.93 °C on DOY 239) and they slightly decreased again from DOY 240 (the end of August). In September, the average temperatures ranged, similar to June, between 18.47 °C and 28.19 °C. Rises in VPD were recorded throughout the entire season, but notable peaks occurred on DOYs 193 and 194 (~3 kPa), 205 (3.24 kPa), and 206 (3.85 kPa). As typical for the region, rain events were few and infrequent; 95.40 mm of precipitation occurred during the growing season of cotton, 37% of which fell during the final phase of the cycle (second half of September) (Figure 2). The total amount of ETc for the growing season was 460 mm.

Table 2 reports the descriptive statistics of the SWP measured during the growing season, per DOY, and for the full dataset. The overall average SWP was −0.43 (±0.29) MPa, the median was −0.33 MPa, and the minimum and maximum values were −1.48 MPa and −0.15 MPa, respectively. The lowest mean values of SWP were detected on DOY 204 (−0.95 MPa). The Supplementary Materials (Table S1) report the descriptive statistics of the S2-Bs used as predictors in this study.

### 3.2. Models’ Evaluation

**Figure 3:**
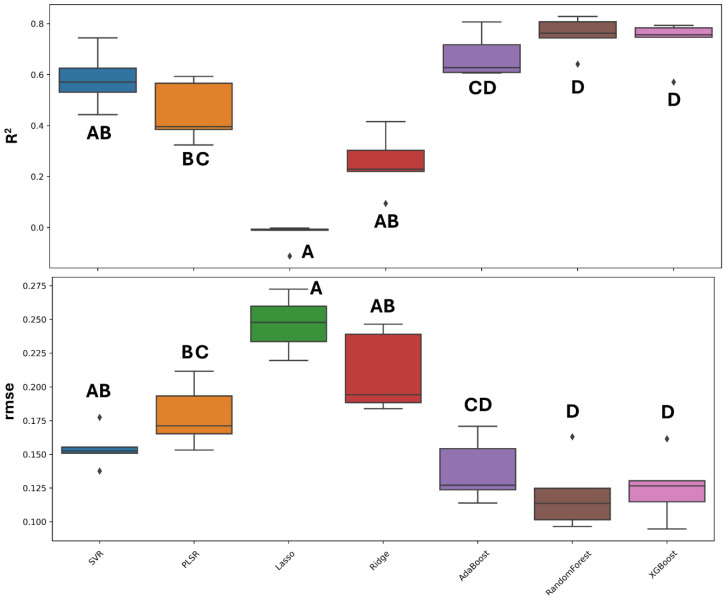
Boxplots showing the distribution of performance parameters for the testing sets from five random splits (total: *n* = 144) of the machine learning models used in this study. The black line within the box represents the median. Different letters denote statistically significant differences among the models at *p* < 0.05, as determined by Tukey’s test.

**Figure 4:**
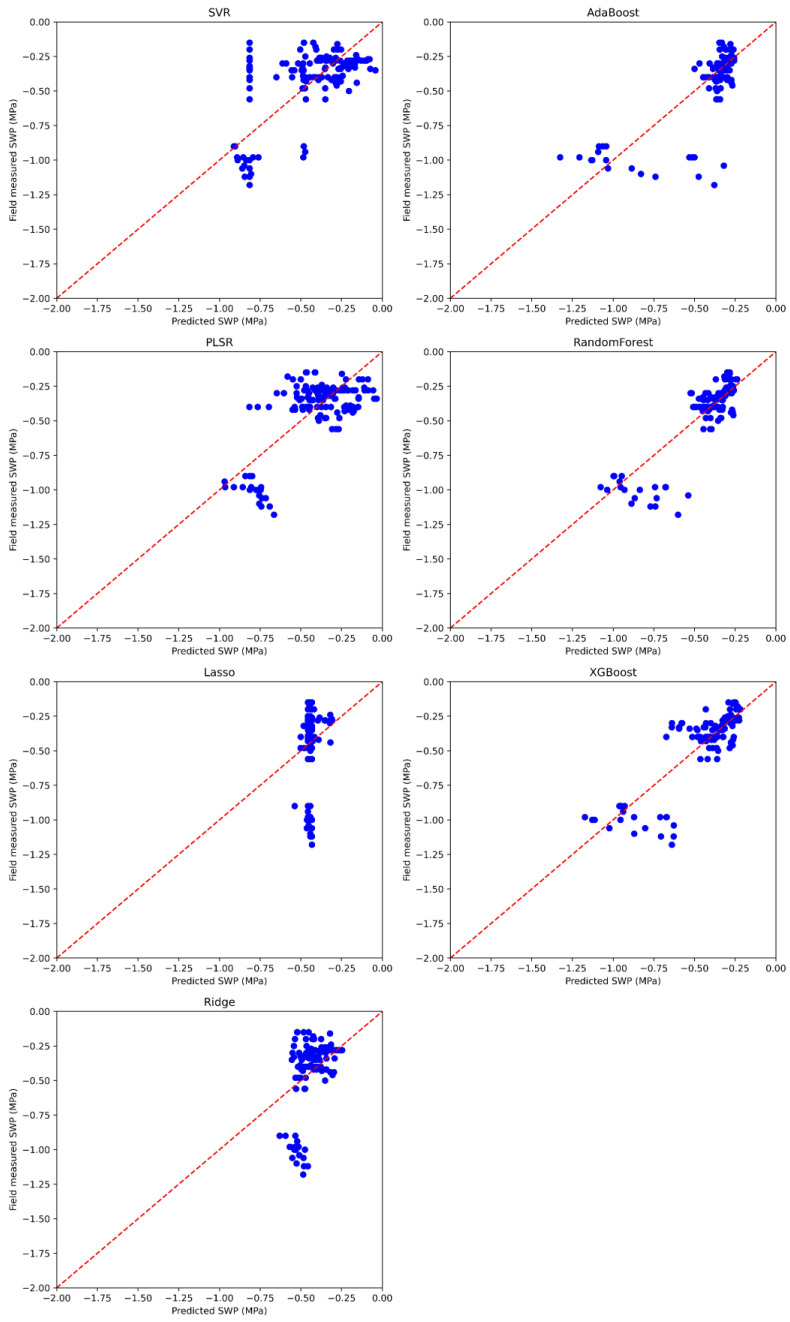
Scatterplots comparing field-measured and predicted stem water potential (SWP; MPa) of cotton for each machine learning model used in this study. The results are based on testing sets from five repeated random splits of the dataset (total: *n* = 144).

The model that yielded the highest average R^2^ was RF (0.75 ± 0.07), and it also achieved the lowest RMSE (0.11 ± 0.02), followed by XGBoost (R^2^: 0.73 ± 0.09, RMSE: 0.12 ± 0.02) and AdaBoost (R^2^: 0.67 ± 0.08, RMSE: 0.13 ± 0.02). The other machine learning algorithms demonstrated lower or even negative performance, for instance, Lasso had an R^2^ of −0.02 (± 0.04) and an RMSE of 0.24 (±0.02). Figure 3 present boxplots illustrating the distribution of the performance metrics of the tested models and the results of Tukey’s test for means comparison. Specifically, while Table 3 reports the average values and their standard deviations for each model, Figure 4 shows the regression plot of the considered models across the five random splits of the dataset. The tree-based algorithms—AdaBoost, RF, and XGBoost—had a significantly higher performance for both R^2^ and RMSE than the other models. Nonetheless, we chose to select the RF model as the best model to apply, due to the higher average R^2^ and lower standard deviation compared with AdaBoost and XGBoost. 

### 3.3. Features’ Importance

**Figure 5:**
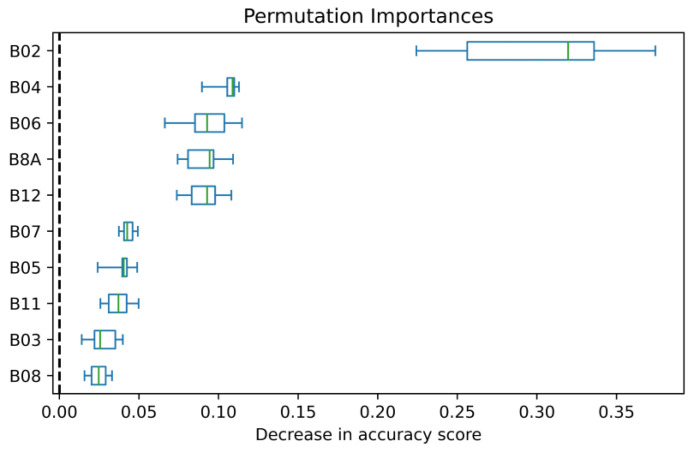
Permutation importance of the features used to predict cotton’s stem water potential using random forest.

**Figure 6:**
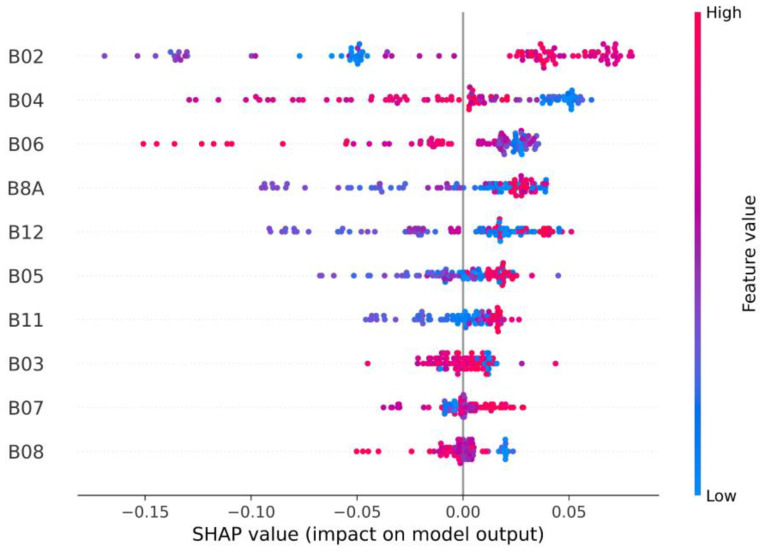
SHAP summary plot showing the importance of the features (displayed in order of importance from top to bottom) and their impact on the model’s output.

The permutation plot (Figure 5) shows that the most important S2-B for the prediction of cotton’s SWP using the RF-based model was B02 (blue region), followed by B04 (red), B06, and B8A (red edge regions). The least important for the prediction of SWP was B08. The SHAP method confirmed the importance of the same bands in this modeling approach (Figure 6); furthermore, the SHAP summary plot shows that higher values of B02 had a positive impact on the RF model’s outcomes. For B04 and B06, low and medium values had a positive impact on the model; in comparison, lower values had a negative impact. In the case of B8A, it seems that high and low values improved predictions, while medium values did not.

**Figure 7:**
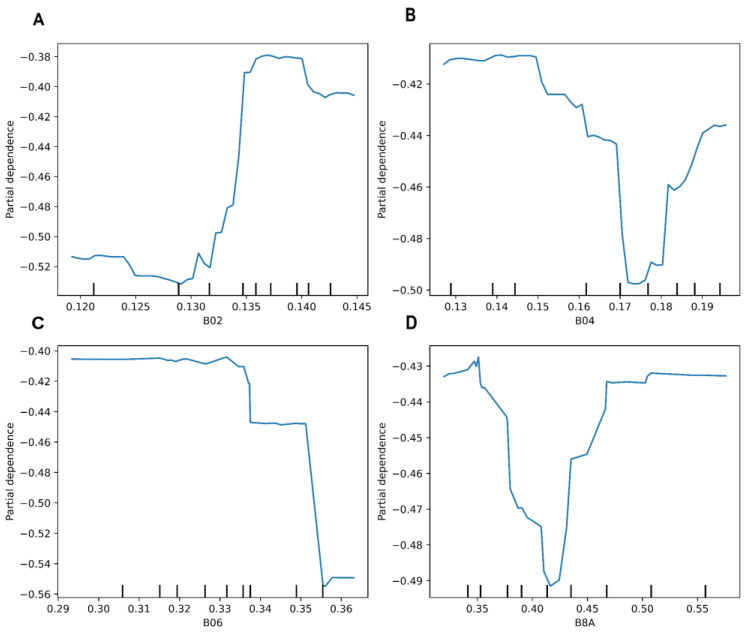
Partial dependence plot of the four most important Sentinel-2 spectral bands ((**A**) B02; (**B**) B04; (**C**) B06; (**D**) B8A) used as predictors with the random forest model.

Figure 7 shows the partial dependence graphs for the four most important S2-Bs in the prediction of cotton’s SWP using the RF model. The partial dependence for the B02 band shows a relatively stable trend with values of around −0.52 up to about 0.130. After this point, there is a sharp increase in the partial dependence, reaching −0.38 at 0.135. This indicates that as the values of B02 increase, the partial dependence increases; as the B02 values increase above 0.130, partial dependency becomes less negative, increasing the predicted SWP. The partial dependence for the B04 range starts to be stable around −0.42 but shows oscillations. The partial dependence decreases sharply from around −0.48 to around 0.165 and then gradually increases to −0.44 at 0.18. These variations could indicate thresholds in B04 that may negatively influence the SWP predictions. The partial dependence for B06 is relatively stable around −0.40 from 0.42 up to about 0.33, after which, there is a sharp decline to about −0.56 at 0.36. This indicates that higher values in B06 may negatively influence the prediction of SWP, and then higher values in B06 are associated with a significant reduction in the prediction of SWP. The partial dependence for B8A shows significant variations, starting at around −0.43, dropping sharply to about −0.49 around 0.42, and then increasing again to −0.43. This variability indicates that the B8A band has a complex impact on the prediction of the SWP.

### 3.4. Predicted Stem Water Potential

**Figure 8:**
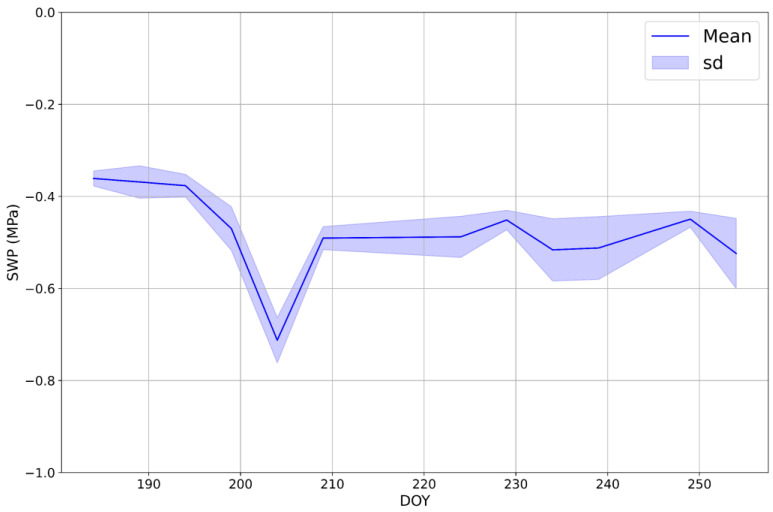
The trend of the stem water potential (SWP; MPa) of cotton simulated by using random forest trained with Sentinel-2 spectral bands from July to mid-September.

The trend of cotton’s SWP was simulated using the RF model and the Sentinel-2 images available for the period from July (the beginning of the development phase) to mid-September (the beginning of the ripening phase) (Figure 8). During the first phase of development, the predicted SWP of cotton remained relatively stable at ~−0.37 MPa and started to drop on DOY 199 (−0.46 ± 0.04 MPa). Thereafter, it decreased markedly to −0.71 ± 0.05 MPa by DOY 204. Subsequently, on DOY 209, the predicted SWP increased to −0.49 ± 0.02 MPa and maintained this level until the latter half of August, except for DOY 229, when it had a slight rise to −0.45 ± 0.02 MPa. On DOY 234, the predicted SWP dropped again to −0.51 ± 0.06 and remained stable until DOY 249, when it rose to −0.45 ± 0.01 MPa. Finally, on DOY 254, a further decrease in SWP was observed, with values falling to −0.52 ± 0.07 MPa.

## 4. Discussion

This research work investigated the application of machine learning techniques for estimating the stem water potential of cotton in the Mediterranean area by using multispectral data from Sentinel-2 imagery. The results provided significant insights into the feasibility of this approach in the efficient monitoring of cotton’s water status, especially in a context affected by global warming and water scarcity such as the regions of the Mediterranean basin.

Among the machine learning algorithms tested, the RF model showed higher performance. This result is in line with findings from research work on other crops and biophysics parameters. For instance, Minaei et al. [59] reported better performance for RF compared with SVR in estimating the leaf nitrogen content of sugarcane at field scale using Sentinel-2 data. Also, the findings of Garofalo et al. [3] highlighted that RF outperformed SVR and linear regression for the estimation of olive’s water status with Planet satellite images. Pôças et al. [60] et al. compared different machine learning algorithms—including RF—to predict grapevine’s predawn leaf water potential in Portugal, showing an R^2^ of 0.77. The robustness of RF in handling non-linear relationships and the complex interactions among the variables typical of remotely sensed data is well documented [61]. In a work from Lin et al. [31], the machine learning-based prediction of SWP was carried out in a different agro-climatic context (Texas, USA). This is a key point, since it could confirm the applicability of the approach, increasing its potential generalization. In our work, we extended this approach by evaluating a broader spectrum of machine learning algorithms (AdaBoost, SVR, Lasso, Ridge, PLSR, RF, and XGBoost) to offer a more comprehensive insight into the potential and limitations of various modeling techniques for this specific application. Lin et al. found better model performance using Sentinel-2 spectral bands rather than vegetation indices. In the present research work, only S2-Bs have been used as predicting variable, without calculating the vegetation indices. Other research has shown that better results can be obtained when using spectral bands as predictors rather than vegetation indices [62,63]. This could be because the use of the reflectance value of all bands in Sentinel 2 allows all information included in the spectrum to be considered [64]. Furthermore, although there are undoubted advantages in using vegetation indices, some problems may arise, such as saturation of the NDVI at high LAI values [65], which may lead to worse performance when using vegetation indices, compared with bands. In this research, techniques for explainable machine learning have been applied to provide insights into mechanisms driving the models’ predictions. The analysis of the importance of the variables showed the predominant role of the visible bands (blue B02 and red B04) and the red edge (B06, B8A) in prediction of the SWP. These results are consistent with previous studies that have demonstrated the sensitivity of these spectral regions to plant water status and stress but provide new insights specific to cotton in the Mediterranean environment [58,66]. The predominance of the blue band (B02) as the most important predictor is particularly relevant from an ecophysiological point of view. This band is known for its sensitivity to changes in the content of ancillary foliar pigments, e.g., carotenoids, which are often associated with water stress, as they act as antioxidants under stress conditions [67,68] In cotton, the accumulation of these pigments in response to various environmental stresses has been documented and represents a crucial photoprotective mechanism under high radiation and water deficit conditions [69,70]. According to the results of partial dependence, the B02 is particularly influential in predicting higher SWP levels when its values are within the range of 0.130 to 0.135; this could be related to the model’s ability to detect specific changes in reflectance corresponding to different water states in the cotton. The second most important band in the RF model was the red band (B04). The B04 band is critical for predicting lower SWP levels around 0.165. These fluctuations might reflect the absorption of red light by vegetation, which is sensitive to changes in plant conditions and thus to their water status; water stress could lead to higher reflectance in the red band [71]. The red edge band (B06) confirmed their usefulness in estimating vegetation and crop conditions, including water status [72,73]. The sensitivity of this spectral region could be related to changes in the chlorophyll content and internal leaf structure that occur in response to water stress [74]. The B06 band is important for determining lower SWP levels when its value exceeds 0.33. The reduction in partial dependence values suggests that this band is effective in detecting water stress conditions, as the red edge is sensitive to variations in chlorophyll content and overall plant health. In cotton growing, the red edge region has been used to monitor drought stress using vegetation indices [30]. The minor importance of the NIR band (B08), on the other hand, is contrary to the literature, as it is usually used for estimating water status [75]. However, in another study by Garofalo et al. on carob tree [35], the NIR region had the lowest importance in predicting the stomatal conductance, a physiological parameter related to plant water status, with RF. This could suggest that with more complex algorithms, the role of the spectral bands as predictors and their importance could depend on the model, the specific crop, the dataset, and the field-related parameters [76,77]. 

## 5. Conclusions and Future Research

The accurate estimation of cotton’s stem water potential using remote sensing and machine learning offers significant opportunities for the implementation of advanced precision irrigation strategies in cotton. High-frequency monitoring of cotton’s water status can drive timely and targeted irrigation interventions, supporting the implementation of deficit irrigation strategies (e.g., regulated deficit irrigation, sustained deficit irrigation) to maximize water use efficiency. The random forest algorithm has been confirmed as one of the most suitable for the detection of water-related crop conditions. Explainable machine learning techniques highlighted the role of the different features in the prediction of cotton’s stem water potential with the random forest model. Despite the promising outcomes, this study has certain limitations that open avenues for future research. As the study was conducted on a single field over one growing season with a relatively small dataset, the findings may be specific to the particular agro-environmental conditions of the study area. This controlled setting allowed for a focused analysis but may limit the generalizability of the results to other regions with different climatic conditions, soil types, or management practices. Future research should aim to validate and enhance the robustness of the proposed models by including multiple fields across diverse geographical locations and extending the study over multiple growing seasons. Expanding the dataset would not only strengthen the statistical power of the models but would also enable the exploration of the temporal dynamics and spatial variability in cotton’s water status. Future research perspectives also include the integration of multi-sensor data, combining spectral information from Sentinel-2 with thermal data (e.g., Landsat 8) or from high-resolution satellite images (e.g., Planet SuperDove images). In addition, it would be useful to assess the economic impact and water-saving potential of implementing this approach on a larger scale. Quantifying the possible reductions in water use and the associated energy costs could provide insights into the practical benefits of a large-scale adoption of the proposed framework.

## Figures and Tables

**Table 1:** Hyper-parameters fine-tuned using Optuna for each model.

Algorithm	Fine-Tuned Hyper-Parameters
AdaBoost	Learning rate; loss; number of estimators
SVR	c; γ; epsilon
Lasso	α; maximum number of iterations
Ridge	α; maximum number of iterations
PLSR	Number of components
RF	Maximum number of features; maximum depth; minimum samples to split an internal node; minimum number of samples of a leaf node after the split; number of estimators
XGBoost	Learning rate; γ; minimum child weight; column sample by tree; subsample; maximum depth

**Table 2:** Descriptive statistics of the stem water potential (MPa) of cotton measured during the season for each day of year (DOY) considered in the study and for the whole dataset (overall). sd = standard deviation.

DOY	Count	Min	Max	Mean	sd	Median
194	16	−0.35	−0.15	−0.26	0.06	−0.27
199	16	−0.56	−0.30	−0.41	0.06	−0.40
204	16	−1.48	−0.18	−0.95	0.31	−1.00
209	16	−0.50	−0.30	−0.38	0.06	−0.40
224	16	−0.40	−0.18	−0.26	0.06	−0.28
234	16	−0.46	−0.16	−0.29	0.07	−0.28
Overall	96	−1.48	−0.15	−0.43	0.29	−0.33

**Table 3:** Performance parameters of the machine learning algorithms used in this study for predicting cotton’s stem water potential, using the Sentinel-2 spectral bands as predictors. sd = standard deviation.

Model	Average R^2^	R^2^ sd	Average RMSE (MPa)	RMSE sd (MPa)
AdaBoost	0.673	0.087	0.137	0.023
Lasso	−0.027	0.047	0.246	0.020
PLSR	0.452	0.119	0.178	0.023
RandomForest	0.756	0.072	0.119	0.026
Ridge	0.252	0.118	0.210	0.029
SVR	0.582	0.112	0.154	0.014
XGBoost	0.730	0.091	0.125	0.024

## Data Availability

The original contributions presented in the study are included in the article/Supplementary Materials; further inquiries can be directed to the corresponding author.

## Supplementary Materials

The following supporting information can be downloaded at: https://www.mdpi.com/article/10.3390/plants13233325/s1, Table S1: Descriptive statistics of the reflectance value of the Sentinel-2 spectral bands used as predictors, per each day of year (DOY) considered in the study, and for the whole dataset (overall). sd = standard deviation.
