# Supplementary material for: Explainable Artificial Intelligence to Predict the Water Status of Cotton (Gossypium hirsutum L., 1763) from Sentinel-2 Images in the Mediterranean Area
Source: Plants (Basel). 2024 Nov 27;13(23):3325. doi: 10.3390/plants13233325 (PMC11644724; doi:10.3390/plants13233325)
Supplement: Supplementary file 1 [file plants-13-03325-s001.zip › plants-3298436-supplementary.pdf]

Table S.1. Descriptive statistics of the reflectance value of the Sentinel-2 spectral bands used as predictors, per each day of year (DOY) considered in the study, and for the whole dataset (overall). sd = standard deviation.

DOY	count	min	max	mean	sd	median
			B02			
194	16	0.134	0.140	0.137	0.002	0.137
199	16	0.140	0.148	0.143	0.002	0.143
204	16	0.126	0.135	0.130	0.002	0.130
209	16	0.135	0.142	0.139	0.002	0.140
224	16	0.132	0.140	0.136	0.002	0.136
234	16	0.118	0.123	0.120	0.002	0.121
overall	96	0.118	0.148	0.134	0.008	0.136
			B03			
194	16	0.172	0.181	0.176	0.003	0.177
199	16	0.174	0.184	0.179	0.003	0.180
204	16	0.172	0.179	0.176	0.002	0.177
209	16	0.176	0.184	0.179	0.002	0.179
224	16	0.172	0.178	0.176	0.001	0.176
234	16	0.158	0.165	0.162	0.002	0.162
overall	96	0.158	0.184	0.175	0.006	0.177
			B04			
194	16	0.188	0.204	0.195	0.004	0.195
199	16	0.178	0.191	0.186	0.003	0.186
204	16	0.170	0.185	0.177	0.004	0.176
209	16	0.153	0.170	0.164	0.005	0.162
224	16	0.137	0.146	0.142	0.003	0.141
234	16	0.124	0.135	0.129	0.003	0.128
overall	96	0.124	0.204	0.165	0.024	0.170
			B05			
194	16	0.335	0.394	0.362	0.022	0.357
199	16	0.366	0.424	0.390	0.018	0.386
204	16	0.409	0.462	0.433	0.016	0.432
209	16	0.446	0.505	0.475	0.021	0.474
224	16	0.559	0.630	0.595	0.021	0.601
234	16	0.585	0.651	0.618	0.020	0.622
overall	96	0.335	0.651	0.479	0.099	0.449
			B06			
194	16	0.332	0.363	0.343	0.011	0.340
199	16	0.319	0.349	0.334	0.012	0.334
204	16	0.335	0.361	0.347	0.011	0.347
209	16	0.315	0.351	0.329	0.015	0.324
224	16	0.306	0.337	0.320	0.014	0.317
234	16	0.293	0.322	0.308	0.013	0.308
overall	96	0.293	0.363	0.330	0.018	0.332
			B07			
194	16	0.263	0.284	0.271	0.009	0.268
199	16	0.253	0.279	0.265	0.012	0.265

204	16	0.245	0.272	0.256	0.012	0.252
209	16	0.221	0.269	0.240	0.019	0.237
224	16	0.197	0.247	0.217	0.021	0.212
234	16	0.184	0.230	0.205	0.020	0.203
overall	96	0.184	0.284	0.242	0.029	0.248
				B08		
194	16	0.242	0.268	0.249	0.009	0.245
199	16	0.238	0.256	0.246	0.007	0.245
204	16	0.239	0.261	0.250	0.008	0.250
209	16	0.237	0.256	0.244	0.008	0.242
224	16	0.236	0.254	0.243	0.007	0.240
234	16	0.214	0.234	0.223	0.008	0.223
overall	96	0.214	0.268	0.243	0.012	0.243
				B8A		
194	16	0.320	0.357	0.338	0.014	0.338
199	16	0.341	0.377	0.360	0.014	0.354
204	16	0.380	0.424	0.405	0.018	0.410
209	16	0.387	0.450	0.424	0.023	0.433
224	16	0.466	0.576	0.524	0.042	0.529
234	16	0.473	0.573	0.529	0.040	0.533
overall	96	0.320	0.576	0.430	0.079	0.413
				B11		
194	16	0.341	0.380	0.360	0.019	0.357
199	16	0.366	0.411	0.389	0.018	0.383
204	16	0.407	0.453	0.430	0.019	0.431
209	16	0.429	0.508	0.469	0.030	0.474
224	16	0.498	0.631	0.581	0.047	0.591
234	16	0.530	0.663	0.613	0.054	0.625
overall	96	0.341	0.663	0.474	0.100	0.445
				B12		
194	16	0.348	0.390	0.368	0.016	0.368
199	16	0.373	0.415	0.393	0.016	0.386
204	16	0.417	0.470	0.444	0.019	0.442
209	16	0.427	0.500	0.470	0.026	0.478
224	16	0.523	0.632	0.588	0.044	0.608
234	16	0.542	0.668	0.614	0.048	0.622
overall	96	0.348	0.668	0.479	0.098	0.454
